# Post-exposure prophylaxis (PEP) efficacy of rifampin, rifapentine, moxifloxacin, minocycline, and clarithromycin in a susceptible-subclinical model of leprosy

**DOI:** 10.1371/journal.pntd.0008583

**Published:** 2020-09-16

**Authors:** Shannon M. Lenz, Jaymes H. Collins, Nashone A. Ray, Deanna A. Hagge, Ramanuj Lahiri, Linda B. Adams

**Affiliations:** 1 IHRC, Inc. Atlanta, Georgia, United States of America; 2 Department of Health and Human Services, Health Resources and Services Administration, Healthcare Systems Bureau, National Hansen’s Disease Programs–Laboratory Research Branch, Baton Rouge, Louisiana, United States of America; 3 Mycobacterial Research Laboratories, Anandaban Hospital, The Leprosy Mission Nepal, Kathmandu, Nepal; Adolfo Lutz Institute of Sao Jose do Rio Preto, BRAZIL

## Abstract

**Background:**

Subclinical infection with *Mycobacterium leprae* is one potential source of leprosy transmission, and post-exposure prophylaxis (PEP) regimens have been proposed to control this source. Because PEP trials require considerable investment, we applied a sensitive variation of the kinetic mouse footpad (MFP) screening assay to aid in the choice of drugs and regimens for clinical trials.

**Methodology/Principal findings:**

Athymic nude mice were inoculated in the footpad (FP) with 6 x 10^3^ viable *M*. *leprae* and treated by gastric gavage with a single dose of Rifampin (SDR), Rifampin + Ofloxacin + Minocycline (SD-ROM), or Rifapentine + Minocycline + Moxifloxacin (SD-PMM) or with the proposed PEP++ regimen of three once-monthly doses of Rifampin + Moxifloxacin (RM), Rifampin + Clarithromycin (RC), Rifapentine + Moxifloxacin (PM), or Rifapentine + Clarithromycin (PC). At various times post-treatment, DNA was purified from the FP, and *M*. *leprae* were enumerated by RLEP quantitative PCR. A regression analysis was calculated to determine the expected RLEP value if 99.9% of the bacilli were killed after the administration of each regimen. SDR and SD-ROM induced little growth delay in this highly susceptible murine model of subclinical infection. In contrast, SD-PMM delayed measurable *M*. *leprae* growth above the inoculum by 8 months. The four multi-dose regimens delayed bacterial growth for >9months post-treatment cessation.

**Conclusions/Significance:**

The delay in discernable *M*. *leprae* growth post-treatment was an excellent indicator of drug efficacy for both early (3–4 months) and late (8–9 months) drug efficacy. Our data indicates that multi-dose PEP may be required to control infection in highly susceptible individuals with subclinical leprosy to prevent disease and decrease transmission.

## Author summary

While multi-drug therapy (MDT) has been successful in decreasing the worldwide prevalence of leprosy, the new case detection rate, or incidence, remains consistent. These circumstances indicate that leprosy transmission is still occurring. Subclinical asymptomatic leprosy infections are considered a leading cause of ongoing transmission. One means to control this source is an effective post-exposure prophylaxis (PEP) regimen that would prevent both subsequent progression to clinical leprosy for the individual and transmission of the disease to others. Therefore, in this study, we used a modified kinetic mouse footpad screening assay and sensitive molecular bacterial enumeration in a susceptible-subclinical mouse model to identify effective potential PEP drug regimens for leprosy. Using these methods, we showed that a single dose PEP regimen is not effective in a susceptible host, and multiple intermittent doses of combination therapies are required. This model could provide useful pre-clinical information for the development of PEP regimens for leprosy.

## Introduction

Despite the global success of multi-drug therapy (MDT), it has been estimated that the difference between observed and expected new cases of leprosy may reach 4 million, indicating potentially large numbers of subclinical infections that could be a source of continuing transmission [1]. One known reservoir of subclinical cases is contacts of leprosy patients, particularly of patients with multibacillary (MB) leprosy. Although not all contacts will go on to develop leprosy, it has been reported that contacts of a MB patient are eight times more likely to develop leprosy compared to the general population [2–3]. In addition, SIMCOLEP modeling studies found that treating subclinical infections among contacts had the greatest impact on leprosy transmission [4]. Thus, an appropriate post-exposure prophylaxis (PEP) regimen for contacts may effectively reduce the incidence of leprosy in endemic countries.

PEP trials are substantial and expensive undertakings that require considerable resources and manpower. Moreover, follow-up of cases is a long-term investment. An ideal chemoprophylactic regimen would be highly effective, easily administered, especially in resource-poor countries, and have no potential side effects since asymptomatic individuals are being treated. Most PEP protocols, therefore, are based on abbreviated regimens with fewer drugs than the current disease treatment [5]. The earliest leprosy PEP trials tested dapsone monotherapy in schoolchildren in Eastern Africa and India [6–7]. However, after the discovery of dapsone resistance and the development of multidrug therapy (MDT), the majority of chemoprophylaxis trials focused on the bactericidal drug, rifampin. One of the first notable trials was performed in the Marquesas Islands in 1988. This study found that population-based administration of single dose rifampin (SDR) was 35–40% effective after 10 years of follow-up [8–10]. More recently, the COLEP study in Bangladesh (2002–2003) found that a contacts-based administration of SDR was 57% effective, particularly in contacts with a low risk of leprosy due to increased physical distance, lack of genetic susceptibility, or decreased bacterial load [11–12].

The mouse footpad (MFP) assay has been instrumental for examining new drugs for leprosy [13]. The “kinetic” MFP assay is particularly beneficial because it can differentiate bacteriostatic from bactericidal drugs [14]. In this assay, groups of mice are treated with drugs early in infection, and drug efficacy is measured by the time lag between treated and untreated mice to reach maximum growth levels. However, because this model relied on an immunocompetent mouse strain, the sensitivity of the assay was limited due to the functioning immune system’s ability to naturally restrict bacterial growth. Additionally, *M*. *leprae* were enumerated by counting acid fast bacilli (AFB), which cannot reliably detect bacterial levels below 10^5^, and maximum growth in an immunocompetent mouse is in the order of 10^6^ bacteria. This further reduced sensitivity and prohibited the determination of early drug effects.

Consequently, we advanced the kinetic MFP assay [14] to increase sensitivity and allow detection of early, as well as later, inhibitory effects of the drugs. Our assay utilizes a low dose *M*. *leprae* infection of athymic nude mice to model susceptible-subclinical contacts, and the RLEP quantitative PCR (qPCR) rather than microscopic counting to enumerate bacilli. We used this model to test the efficacy of single dose and multi-dose regimens of rifampicin, rifapentine, moxifloxacin, minocycline, and clarithromycin as potential leprosy PEP.

## Methods

### Ethics statement

Experiments were performed in accordance with the United States Public Health Service Policy on the Humane Care and Use of Laboratory Animals. The National Hansen’s Disease Programs Institutional Animal Care and Use Committee (Assurance #D16-00019 [A3032-01]) reviewed and approved all protocols.

### Maintenance of viable *M*. *leprae* inoculum

*M*. *leprae*, strain Thai-53, is maintained through serial passage in athymic nude mice (Envigo) to maintain maximum viability [15–16]. *M*. *leprae* were harvested from the footpads (FP), stored at 4⁰C, and used within 24 hours for inoculation.

### Murine model and infection

Athymic nude mice (Envigo) were inoculated in both hind FP with 6 x 10^3^ *M*. *leprae*. Mice were treated by gastric gavage (0.2ml) with vehicle (hydroxypropyl-β-cyclodextrin, 100mg/ml) or vehicle plus drug(s) in various combinations. Drugs administered were rifampin (10mg/kg), ofloxacin (150mg/kg), minocycline (25mg/kg), rifapentine (10mg/kg), moxifloxacin (150mg/kg), and clarithromycin (100mg/kg). These drug dosages are equivalent human adult dose per weight ratios (Table 1), except for clarithromycin, which is the pediatric dose equivalent. DNA was extracted from the FP at various time points post-treatment as previously described [17], and *M*. *leprae* were enumerated by RLEP qPCR [18].

**Table 1:** Drugs used in studies.

Drug	Human Dose	Equivalent Mouse Dose	REFERENCES
Rifampin	600mg	10mg/kg	[32]
Ofloxacin	400mg	150mg/kg	[33]
Minocycline	100mg	25mg/kg	[34]
Moxifloxacin	400mg	150mg/kg	[35]
Clarithromycin	330mg	100mg/kg	[36–37]
Rifapentine	600mg	10mg/kg	[38]

### Single dose studies

*M*. *leprae*-infected mice were given a single dose of rifampin (SDR), a single dose of rifampin + ofloxacin + minocycline (SD-ROM), or a single dose of rifapentine + moxifloxacin + minocycline (SD-PMM) via gastric gavage. For each group, FP were harvested just prior to the appropriate drug administration (T0). Remaining FP were harvested at two, four, six, eight, nine, and/or ten months post-treatment.

### PEP++ drug study

*M*. *leprae*-infected mice received three once-monthly doses of rifampin + moxifloxacin (RM), rifampin + clarithromycin (RC), rifapentine + moxifloxacin (PM), or rifapentine + clarithromycin (PC) via gastric gavage. T0 mice were harvested just prior to the administration of the first drug treatment. FP were harvested at one, three, six, and nine months after the completion of all three treatments.

### Statistical analyses

GraphPad Prism 8.0.2 and SigmaPlot 11.0 were used to perform Mann Whitney Rank Sum analyses to compare the different groups and within a group. The untreated control group was used to develop a regression model of *M*. *leprae* growth and calculate expected numbers of bacilli, if the drug intervention killed 99.9% of bacilli present at T0, at early and late timepoints. Data was considered significant at P<0.05.

## Results

### Single dose PEP regimens are unable to control *M*. *leprae* growth

We initially used our murine model to determine the efficacy of single dose drug regimens (SDR, SD-ROM, and SD-PMM). In the first study, the drugs were administered at different levels of initial infection of ~10^2^ (3.69 x 10^2^ ± 2.23 x 10^2^), ~10^3^ (9.66 x 10^2^ ± 3.89 x 10^2^), or ~10^4^ (1.21 x 10^4^ ± 7.46 x 10^3^) bacilli per FP. In order to achieve the different initial infection levels, all mice were inoculated with 6 x 10^3^ *M*. *leprae* at the same time, but the treatments were staggered at 1 day, 1 month, and 2 months post-inoculation to allow for different levels of initial infection. Two of these infection levels, 10^2^ and 10^3^, were considered subclinical infections at the beginning of the experiment as they would have been undetectable by traditional acid-fast counting [19]. Each group was harvested around the order of 10^9^ to 10^10^ bacilli.

**Fig 1:**
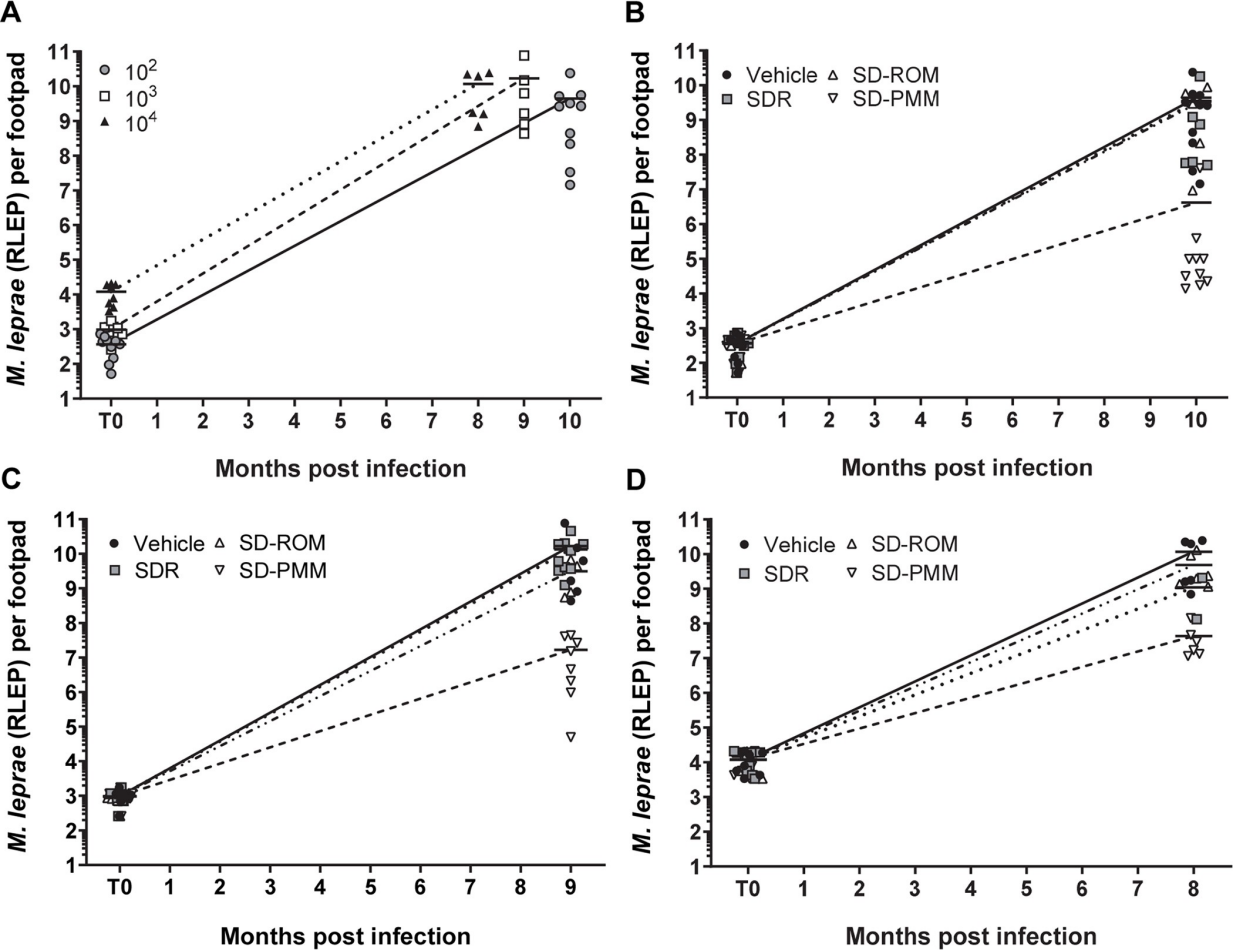
Single dose PEP regimens are unable to control *M*. *leprae* growth regardless of baseline levels of bacilli. Athymic nude mice were infected in both hind footpads with 6 x10^3^ *M*. *leprae*. Mice were treated with single dose rifampin (SDR), single dose ROM (rifampin, ofloxacin, minocycline), or single dose PMM (rifapentine, moxifloxacin, minocycline) at 1 day, 1 month, or 2 months post-inoculation. *M*. *leprae* were enumerated by RLEP qPCR. (A) Comparison of growth of the vehicle groups at all initial infectious doses (10^2^, 10^3^, and 10^4^ bacilli). Comparison of growth at (B) 10^2^ bacilli, (C) 10^3^ bacilli, and (D) 10^4^ bacilli. Bars represent the mean for each group.

All of the vehicle groups had significant growth compared to their respective T0 levels (P ≤ 0.001 for each) (Fig 1A) confirming that the initial inoculum was viable. As expected, a lower initial infectious dose required longer to reach peak growth. An initial infection of 10^4^ *M*. *leprae* required 8 months to reach peak levels, whereas 10^3^ and 10^2^ required 9 and 10 months, respectively (P = 0.435). The average generation time for *M*. *leprae* in all groups was 12.56 ± 0.59 days.

Regardless of the bacterial load at treatment, neither SDR nor SD-ROM had significantly different levels of *M*. *leprae* growth compared to the vehicle (Fig 1B–1D). However, there was a significant delay in bacterial growth in the SD-PMM treatment group when drug administration occurred at an initial infection of either 10^2^ (Fig 1B) or 10^3^ (Fig 1C) (P < 0.001). In contrast, while SD-PMM was still effective at an infectious dose of 10^4^, it was not as significantly different compared to the vehicle (P = 0.002; Fig 1D). This indicates that in this immunocompromised population SD-PMM was more effective when the level of infection or bacterial load is lowest. However, significant growth does still occur.

**Fig 2:**
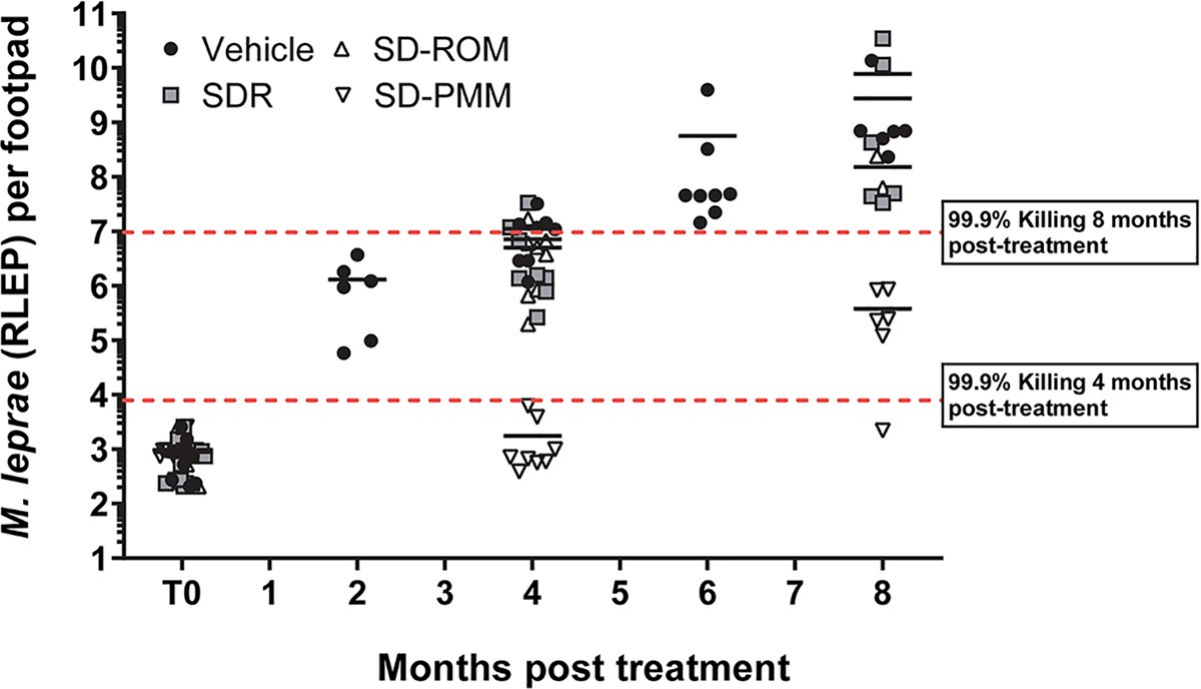
Early and late efficacy of single dose PEP regimens against *M*. *leprae*. Athymic nude mice were infected in both hind footpads with 6 x10^3^ *M*. *leprae*. Mice were treated with single dose rifampin (SDR), single dose ROM (rifampin, ofloxacin, minocycline), or single dose PMM (rifapentine, moxifloxacin, minocycline). *M*. *leprae* were enumerated by RLEP qPCR at two, four, six, and eight months post treatment. Bars represent the mean for each group. A regression analysis was calculated to determine the expected RLEP value if 99.9% of the bacilli were killed after the administration of the single dose regimen (r^2^ = 0.933). The 99.9% kill line at 4 months was 7.94 x 10^3^ bacilli, and the 99.9% kill line at 8 months was 9.55 x 10^6^ bacilli.

In the second study, we examined the early dynamics of the different single dose regimens (SDR, SD-ROM, SD-PMM). We compared growth within each drug group to a subclinical initial infection level of ~10^3^ (8.92 x 10^2^ ± 7.19 x 10^2^) bacteria (T0). Using the control data, we developed a log-linear regression model (r^2^ = 0.933) of the growth for this specific *M*. *leprae* inoculum in nude MFP. We then used this model to determine the expected *M*. *leprae* growth (RLEP) value at both four and eight months post-treatment, if 99.9% bacilli (compared to the untreated control) were initially killed by the drug treatment. Based on this model, the expected RLEP values were 7.94 x 10^3^ bacilli at four months post-treatment, and 9.55 x 10^6^ bacilli at eight months post-treatment. At four months post-treatment, the means of the vehicle (1.11 x 10^7^ ± 9.93 x 10^6^), SDR (7.17 x 10^6^ ± 1.13 x 10^7^), and SD-ROM (5.05 x 10^6^ ± 5.46 x 10^6^) groups were all above the expected value indicating that the single dose treatment did not kill 99.9% of the initial infectious dose (Fig 2). This trend continued at eight months post-treatment with all three groups reaching bacilli levels of 10^8^ to 10^9^.

In contrast, the means of SD-PMM were below the expected RLEP values at both four months (1.75 x 10^3^ ± 2.14 x 10^3^) and eight months (3.79 x 10^5^ ± 3.68 x 10^5^) post-treatment. Thus, SD-PMM is able to effectively kill 99.9% of the bacilli after the administration of the drug combination. However, growth does occur between four months and eight months post-treatment reaching levels of ~10^5^ bacilli, which is above subclinical levels of infection in our model. This indicates that SD-PMM is unable to completely control bacterial growth for an extended period of time as the organisms not killed by the single treatment are now multiplying. Therefore, this second study confirms the findings of the first study in that even at low bacterial levels, a single dose treatment of a combination of drugs is ineffective in a susceptible host.

### Three doses of rifampin/rifapentine-containing drug combinations are able to control bacilli growth in a highly susceptible mouse model

**Fig 3:**
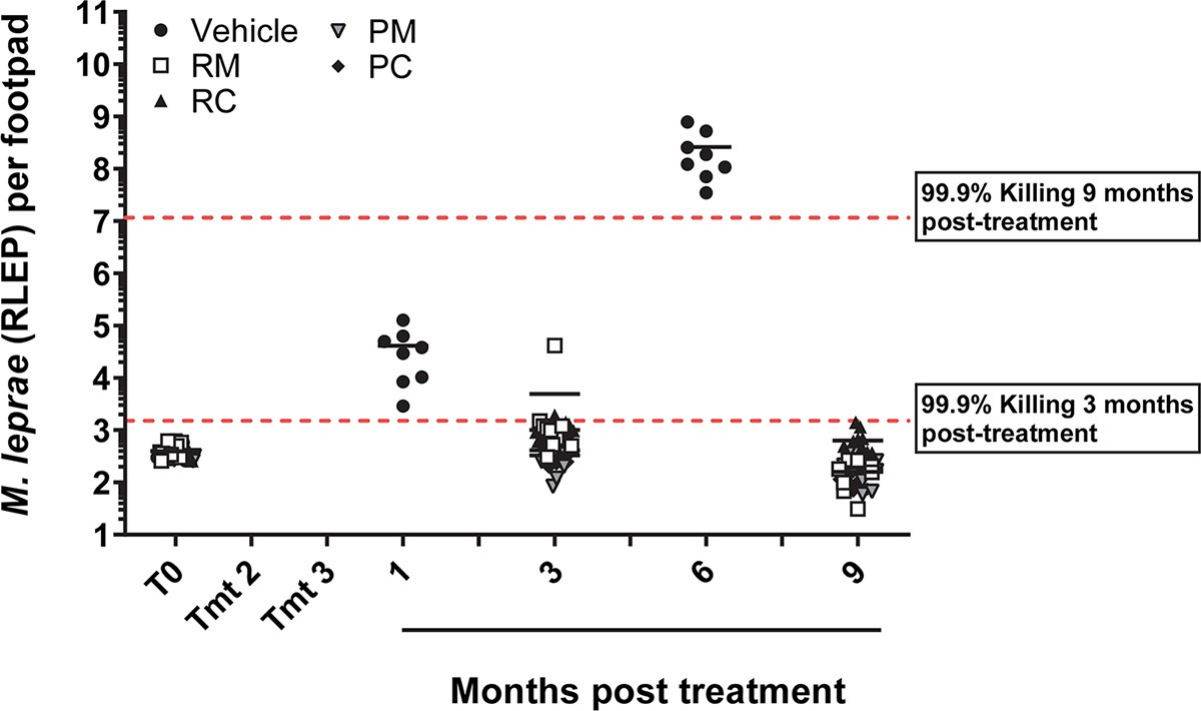
Multi-dose PEP regimens control bacterial growth. Athymic nude mice were infected in both hind footpads with 6 x10^3^ *M*. *leprae*. Mice were treated with three once-monthly doses of rifampin/moxifloxacin (RM), rifampin/clarithromycin (RC), rifapentine/moxifloxacin (PM), or rifapentine/clarithromycin (PC). *M*. *leprae* were enumerated one, three, six, and nine months post treatment completion. Bars represent the mean for each group. A regression analysis was calculated to determine the expected RLEP value if 99.9% of the bacilli were killed after the administration of the three dose regimen (r^2^ = 0.998). The 99.9% kill line at 3 months was 1.51 x 10^3^ bacilli, and the 99.9% kill line at 9 months was 1.16 x 10^7^ bacilli.

The final drug study looked at the efficacy of the proposed PEP++ drug regimen of three once-monthly doses of RM and RC [20]. We also compared RM and RC to PM and PC to determine if there was a significant difference between rifampin and the longer-lasting rifapentine [21]. Using the aforementioned regression model, we calculated the expected bacilli levels for three and nine months post-treatment if 99.9% of the bacteria were killed after completion of the drug regimens. The three month expected value was 1.51 x10^3^ bacilli, and the 9 month expected value was 1.16 x 10^7^ bacilli (r^2^ = 0.998). At three months post-treatment, the means for three out of four treatment groups (RC, PM, & PC) were below the 99.9% killed line (Fig 3), and the RM group was just slightly above it (4.94 x 10^3^ ± 1.28 x 10^4^). This demonstrates that all four of the antibiotic combinations are effectively killing the majority of the initial bacterial load. Additionally, all four groups are well below the expected values at 9 months indicating that all four groups are equally able to control bacterial growth up to 9 months after completion of treatment.

## Discussion

Testing new drugs for efficacy against *M*. *leprae* is a tedious and time-consuming process. The bacteria do not grow on laboratory medium, and in a host *M*. *leprae* grow very slowly with a generation time of 12–14 days. We and others have developed various metabolic, staining, and molecular protocols to determine bacterial viability and have successfully applied these assays for short-term *in vitro* drug screening assays against non-replicating bacteria [reviewed in 19]; however, *M*. *leprae* growth assays remain long-term endeavors. Second, measurement of growth is traditionally determined by counting AFB. This technique has rather poor sensitivity requiring bacterial numbers to reach close to 10^5^ bacilli for reliable determination of growth. Moreover, dead *M*. *leprae* remain in the tissues for months to years, and they are indistinguishable from live *M*. *leprae*. Therefore, even with a highly effective drug regimen, one must wait for the survivors to reach a level substantially higher than the inoculum to be able to differentiate them from bacteria that were killed. Third, the viability of the inoculum could only be assured at the completion of the experiment, i.e. *M*. *leprae* controls grew appropriately; as a result, many experiments were performed using *M*. *leprae* preparations of poor initial viability. Therefore, the objective of this study was to develop a simpler model for examining new drugs or drug regimens against low level *M*. *leprae* infection that could provide useful information for the development of post-exposure prophylactic regimens for leprosy.

In our model, immunocompromised athymic nude mice were infected with a low dose of *M*. *leprae* bacilli to model subclinical infection in a susceptible host. Using an immunosuppressed mouse with no cell-mediated immune response to *M*. *leprae* increases sensitivity of the assay [22–23], removes any contribution of the host immune system toward limiting bacterial growth, and allows measurement solely on the effect of the tested drug against *M*. *leprae*. Additionally, it mimics a "worst-case scenario" that could be seen in human patients i.e. those likely to develop lepromatous leprosy [24–25]. This is a high standard to set for a drug evaluation assay, but if the drug is effective here it should also be effective in immunocompetent mice and arguably would be the best potential candidate for clinical trials. We then measured the efficacy of the PEP regimens using RLEP qPCR [17–18; 26], which is extremely sensitive (~30 bacilli per specimen) and can report actual bacterial numbers in terms of DNA measured against a standard curve, where AFB counting could only report “no growth.” These parameters, along with our highly viable *M*. *leprae* inoculum [15–16] enabled detection of both early and long-term drug efficacy.

We first examined the commonly recommended PEP protocol of SDR and compared it to two other single dose treatments, SD-ROM and SD-PMM (Figs 1 and 2). Significant bacterial growth occurred for both the SDR and SD-ROM groups early in infection. SD-PMM, in contrast, delayed *M*. *leprae* growth for 4 months. SD-PMM contains rifapentine, a long-lasting derivative of rifampin with similar bactericidal activity [21]. Moxifloxacin has also shown better efficacy than ofloxacin against *M*. *leprae* [27–28]. Interestingly, at the lowest initial bacterial loads, the SD-PMM group showed a better growth delay compared to higher infection levels. This finding concurs with what has been seen in human studies suggesting that PEP may be most effective in contacts with lower bacillary loads [11–12; 29].

We also tested the efficacy of the proposed PEP++ regimen of three once-monthly doses of RM for adults and RC for children [20], along with a PM and PC regimen (Fig 3). All four of these regimens delayed growth of *M*. *leprae* for greater than nine months post-treatment indicating a bactericidal effect. Multiple doses may be more effective due to the unique metabolism and slow growth of *M*. *leprae* [30]. A single dose of even a highly effective drug or drug combination, as with SD-PMM above, would not likely kill every bacterium as the bacterial population contains members at various stages of growth and metabolic activity. While an immunocompetent individual’s immune system may be able to compensate for the reduced killing from a single dose, an anergic, i.e. LL, individual may be incapable.

In their initial report, Mieras *et*. *al* [20] proposed that the best combination of PEP++ would be RM for adults and RC for children. More recently, the European Medicines Agency (EMA) has recommended that fluoroquinolones, including moxifloxacin and ofloxacin, should be restricted to second line treatments due to potential side effects [31]. Thus, the use of moxifloxacin in any global prophylaxis regimen may be restricted. However, based on our findings, RC may be a viable alternative to RM for use as PEP in all contacts regardless of age. While RC was slightly less effective than the other PEP++ combinations, it is important to note that we used the pediatric clarithromycin dosage in our study. Since this lower dose was still effective in combination with either rifampin or rifapentine, it is reasonable to assume that the higher adult dosage would be just as or more effective at controlling the bacterial growth.

In conclusion, our modified kinetic MFP assay, which incorporates the athymic nude mouse, a molecular bacterial counting method, and a highly viable *M*. *leprae* inoculum, presents a straightforward assay whereby one can determine PEP efficacy in a susceptible, subclinical model of leprosy. Both early (2–4 months) and late (8–9 months) effects can be examined. Of the single dose regimens, SD-PMM showed strong early activity while neither SDR nor SD-ROM were effective. The multi-dose, multi-drug regimens showed activity both early and late in infection. Therefore, our data suggests that it would be prudent to consider the use of multi-dose PEP for chemoprophylaxis of susceptible individuals.
